# Renal fibrosis progression following partial unilateral ureteral obstruction: mechanisms and therapeutic insights

**DOI:** 10.1007/s00345-025-05580-x

**Published:** 2025-04-17

**Authors:** Yomna Khater, Nashwa Barakat, Ahmed Shokeir, Alaa Samy, Gamal Karrouf

**Affiliations:** 1 Medical Experimental Research Centre, Faculty of Medicine, Mansoura University, Mansoura, 35516 Egypt; 2 Urology and Nephrology Center, Mansoura University, Mansoura, 35516 Egypt; 3 Centre of Excellence of Genome and Cancer Research, Faculty of Medicine, Mansoura University, Mansoura, 35516 Egypt; 4 Department of Surgery, Anesthesiology, and Radiology, Faculty of Veterinary Medicine, Mansoura University, Mansoura, 35516 Egypt

**Keywords:** Partial unilateral ureteral obstruction, Treatment, PUUO, Fibrosis

## Abstract

Ureteral obstruction, a common clinical condition, is associated with various renal disorders affecting all age groups and can lead to permanent renal damage. In Partial unilateral ureteral obstruction (PUUO), increased ureteral pressure, and decreased glomerular filtration rate (GFR) in the affected kidney cause cellular and molecular abnormalities, which ultimately lead to renal fibrosis if untreated. While removing the obstruction (RUUO) is essential to prevent long-term damage, additional therapeutic approaches may be necessary to fully restore kidney function. The PUUO model is used to induce renal fibrosis, primarily characterized by tubular injury resulting from obstructed urine flow. PUUO in rodents can simulate human chronic obstructive nephropathy at an accelerated rate. Although alleviating the obstruction can reduce immediate symptoms, it is often insufficient to reverse established fibrosis, emphasizing the need for adjunctive therapies. The renal response to RUUO depends on factors like the obstruction’s duration and severity, as well as the contralateral kidney's functional state.

## Introduction

Ureteral obstruction is a prevalent renal disorder that can affect individuals of all ages. When left untreated, persistent obstruction can lead to chronic kidney injury. The UUO-obstructed kidney exhibits interstitial expansion, hypertrophy, tubular dilation, proximal tubular mass loss, leukocyte infiltration, hydronephrosis, and tubular epithelial apoptosis. These changes stem from molecular processes such as hemodynamic alteration from mechanical stretching, oxidative stress, and inflammation, collectively leading to progressive fibrosis. However, if detected early, these effects can be managed or even reversed [1]. In the case of Partial unilateral ureteral obstruction (PUUO), pressure increases within the affected ureter, reducing blood supply and consequently lowering the glomerular filtration rate (GFR). This scenario prompts cellular and molecular abnormalities that often progress to fibrosis if left unresolved, eventually causing lasting kidney damage. Studies on UUO emphasize the critical importance of timely removal UUO to prevent severe damage to the obstructed kidney [2].

The kidney’s response to PUUO varies based on factors such as obstruction duration, severity, patient age, ureteral compliance, and the health of the opposite kidney during obstruction. While research has focused on renal hemodynamics, function, and injury after RUUO, less is known about the kidney’s intrinsic repair mechanisms post-relief. Prolonged UUO generally leads to irreversible fibrosis and apoptosis, whereas short-duration obstruction (less than 24 h) is often fully reversible. Following brief UUO, levels of aquaporin channels, sodium transporters, and GFR tend to normalize within two weeks, and the presence of macrophages in the affected kidney also declines within four weeks [3].

Despite these findings, evidence suggests that UUO alone may not fully restore renal function and histopathology, and that additional drug therapies may be necessary. Post-obstruction, free radicals can continue to form, reducing renal blood flow and contributing to ongoing ischemia. PUUO, combined with complementary therapies, is thus crucial for promoting kidney repair, balancing cell loss and proliferation, and mitigating further injury. Various treatment strategies have been proposed to enhance kidney recovery following PUUO in both animal models and human studies [4].

## Causes of ureteral obstruction

Various congenital causes of ureteral obstructions include strictures, ureteroceles, retrocaval ureters, obstructing megaureters, and prune-belly syndrome. Neoplastic causes may involve primary ureteral carcinoma and endometriosis, while miscellaneous factors can include retroperitoneal fibrosis and metastatic carcinoma. Inflammatory conditions such as tuberculosis, schistosomiasis, amyloidosis, and ureteritis cystica may also lead to obstruction. Other contributing factors encompass trauma, pelvic lipomatosis, aortic aneurysms, urinomata, radiofrequency ablation, and pregnancy. Understanding these causes is essential for developing effective interventions and treatments for patients suffering from obstructive uropathy [5].

## Mechanisms of obstructive uropathy

Laboratory animals are frequently employed as models for studying UUO, which can manifest as either complete or partial obstruction, simulating the obstructive uropathy experienced by humans. Several biochemical and molecular factors, such as the oxidative stress and Renin-Angiotensin System (RAS), contribute to the pathophysiology of parenchymal kidney damage in PUUO. Thus, the administration of protective medications is vital in safeguarding the kidneys against the damaging effects of obstruction. Obstructive uropathy refers to the anatomical or functional obstruction of urine flow anywhere in the urinary tract, while obstructive nephropathy results from this obstruction leading to renal damage, whether functional or anatomical. In PUUO, the stationary urine flow causes the rise in ureteral pressure transmitted back to kidney that causes secondary renal vasoconstriction and lead to decrease glomerular blood flow which may lead to ischemia [6].

### RAS activation and mechanical stretching

In the UUO model, the hydrostatic pressure rises due to stagnant urine flow, first dilatation of collecting ducts, and transmission back to proximal and distal tubules. Reduced GFR and mechanical stretching damage to tubular epithelial cells are outcomes of this increase in proximal tubule pressure. Renal Blood Flow (RBF) may rise momentarily in the early stages of UUO, but long-term obstruction eventually results in decreased RBF and renal vasoconstriction, which may result in ischemia [7].

### Apoptosis of tubular epithelial cells

Apoptosis and damage to tubular epithelial cells are outcomes of the first mechanical straining. This apoptosis is also influenced by the release of pro-apoptotic soluble substances and oxidative stress. Tubular cell apoptosis was seen three days after UUO, and its severity rises with time [8].

### Oxidative stress

Oxidative stress is a major factor in the development of kidney injury in the UUO model, starting in early stages. Following UUO, lipid peroxidation markers include malondialdehyde (MDA), superoxide anion generation, and NAD(P)H oxidase (Nox) protein levels are enhanced along with an increase in ROS levels. Additionally, the antioxidant enzymatic system is compromised; at 14 days after UUO, lower levels of catalase and superoxide dismutase were observed. The transcription factor Nuclear Factor (Erythroid-derived 2)-Like 2 (Nrf2), which starts the transcription of antioxidant response elements, is also present in lower amounts. RAS activation and elevated Nuclear Factor κB (NF-κB) may downregulate Nrf2 expression in the UUO model, despite the fact that Nrf2 normally translocates to the nucleus to activate antioxidant enzymes under oxidative stress. In addition to suppressing Nrf2, RAS activation probably causes an increase in ROS production through Nox. At the same time, it promotes the production of factors that drive leukocyte migration and the inflammatory response, such as NF-κB, Tumor Necrosis Factor α (TNF-α), and Transforming Growth Factor β1 (TGF-β1) [9].

### Inflammation

The Wnt/β-catenin pathway controls RAS genes, and activation of RAS triggers fibrosis by triggering the Wnt/β-catenin and TGF-β/Smad signaling pathways. Fibrosis further increases RAS, which in turn continues to activate the TGF-β/Smad and Wnt/β-catenin pathways, resulting in a vicious cycle. Snail1, Twist, Matrix Metalloproteinase 7 (MMP7), Plasminogen Activator Inhibitor 1 (PAI-1), and Fibroblast Specific Protein 1 (FSP-1) are target proteins of the Wnt/β-catenin signaling cascade [10].

### Leukocyte migration

Exosomes, chemokines, and cytokines are released by parenchymal, endothelial, and tubular epithelial cells in response to kidney damage. Beginning on the first day of UUO and continuing for the duration of the obstruction period, this starts the recruitment of leukocytes, mainly neutrophils, T-lymphocytes, and macrophages, to the tubulointerstitium. In the UUO model, inflammation is exacerbated by the accumulation of macrophages in the renal cortex. Increased level of TNF-α, which attract monocytes and macrophages to the tubulointerstitium, drive this migration, which is aided by the production of adhesion molecules such selectins. These infiltrating leukocytes then release TNF-α and TGF-β1, which intensifies the inflammatory response even more [11].

### Fibrosis

The degree of renal fibrosis, a common last stage that leads from chronic kidney disease (CKD) to end-stage renal disease, is directly correlated with decline in kidney function. It may be possible to slow or stop the course of CKD by inhibiting renal fibrosis. Tissue fibrosis and scarring are caused by the buildup of extracellular matrix (ECM), and the terms "fibrosis," "sclerosis," and "scarring" are sometimes used interchangeably. Fibroblasts are the primary contributors to the creation of tubulointerstitial ECM, whereas injured tubular cells contribute to a thicker tubular basement membrane that is predominantly made of collagen type IV [12]. Glycosaminoglycans and other forms of collagen also make up the extracellular matrix. There are no discernible alterations in kidney glomeruli during 21 days of tubular atrophy, which is more closely linked to renal failure and interstitial fibrosis than glomerular damage. With notable tubulointerstitial fibrosis seen in the renal cortex, there is a close link between the advancement of fibrosis and progressive tubular epithelial cell loss. Notably, neonatal rodents may have more severe fibrosis than adults. Renal fibrosis and tube damage after restricted urine flow can be studied using the UUO model, which accurately simulates human chronic obstructive nephropathy. This model's capacity to remove the obstacle and evaluate what happens next is one of its main advantages [7].

## Experimental procedure of UUO

The UUO model is extensively utilized to investigate obstructive nephropathy. This procedure involves ligating the ureter, typically the left one, with silk thread, resulting in the affected kidney being referred to as the Obstructed Kidney (OK). Male animals are often chosen for these studies to avoid complications from female reproductive organs during surgery. The recommended anesthesia to minimize mortality during the procedure is isoflurane/oxygen. It is essential to keep the animal's body temperature with a heated surface and utilize a high-quality binocular microscope for the surgery [13].

### Animal models of PUUO

Various animal species, including rats, opossums, rabbits, pigs, dogs, sheep, and monkeys, have been employed to create models of PUUO using diverse approaches. Early studies laid the foundation for understanding PUUO through different animal models [14].

#### Antenatal models of PUUO

By encircling the ureter with a silastic tube between days 70 and 75 of pregnancy, Tanagho and associates created the first PUUO model in fetal lambs. As early as 1960, a model of inbred unilateral congenital hydronephrosis in rats was developed, and it showed that congenital hydronephrotic rats had substantially higher pelvic pressure as normal rats. This implied a blockage at the ureteropelvic intersection. Subsequent research revealed that the blockage in male rats happened between the middle and upper thirds of the ureter, but in female rats it happened in a slightly different place [14].

#### Newborn models of PUUO

PUUO model was developed in newborn guinea pigs by encasing the ureter in a plastic tube. This method, employed by Taki and Chevalier [15], was notable because nephron differentiation in these animals is complete at birth. However, the fixed internal diameter of the plastic tube makes long-term kidney effects challenging to interpret. Similarly, Josephson et al. [16] created a PUUO model in newborn rats by embedding a ureter segment into the psoas muscles. In this model, glomeruli in the outer quarter of the renal cortex were still undergoing development, analogous to the second or third trimester in human fetuses. The resulting dilation observed in this model appeared early but did not progress, suggesting it mimics mild congenital hydronephrosis.

#### Adult models

In adult animals, a technique for creating PUUO was first described for dogs, where the ureter was embedded in a psoas muscle tunnel. This approach was later adapted by Djurhuus and colleagues to establish PUUO in adult pigs. Another method involved placing a 3-French ureteral catheter along the ureter in dogs, followed by tying a silk ligature around it and withdrawing the catheter. This technique was also successfully applied in monkeys. More recently, a specially designed polypropylene obturator was utilized to induce PUUO in dogs, demonstrating the ongoing evolution of methods for studying this condition in various animal models [14].

## Treatment of PUUO

Current research is focused on therapeutic strategies to address renal fibrosis caused by ureteral obstruction. Although relieving the obstruction may seem beneficial, it often fails to halt kidney damage progression [17]. Studies have shown that obstruction release does not reverse nephron loss or improve tubular cell proliferation, despite normalization of glomerular filtration rates. Furthermore, TGF-β1 levels may continue to rise even after the obstruction is removed, indicating the need for pharmacological treatments to help delay or prevent kidney function loss [18].

### Pharmaceuticals with fibrosis-ameliorating activity in UUO models

#### Aliskiren

Aliskiren inhibits the renin-angiotensin system (RAS) by blocking renin directly, reducing angiotensin II production. Studies by Wang et al. [19] found it prevents downregulation of aquaporin AQP2 in obstructive kidney models (UUO-OK) and reduces inflammatory markers like TGF-β1 and TNF-α. When combined with mizoribine, it showed enhanced effectiveness by further reducing markers such as CD68, interstitial volume, α-SMA-positive cells, and inflammation-related genes.

#### Colchicine

Known for anticancer properties, Colchicine reduced apoptosis in UUO-OKs, inhibiting microtubule polymerization, which plays a role in fibrosis reduction. It decreased markers like acetylated-α-tubulin, TGF-β1, and caspases, thus decreasing fibrosis through possible inflammatory cell reduction [20].

#### Empagliflozin

This SGLT-2 inhibitor and antidiabetic drug lowered creatinine and urea levels and reduced markers such as TGF-β1 and NF-κB in UUO-OKs. It also increased levels of Klotho, a protective protein, suggesting inhibition of the Toll-like receptor 4 (TLR4)/NF-κB pathway, which could play a role in fibrosis suppression [21].

#### Fimasartan

An angiotensin II receptor blocker, Fimasartan reduced oxidative stress and fibrosis markers in UUO-OKs, affecting pathways like Nrf2 and lowering recative oxygen species (ROS) production [22].

#### Metformin

Widely used for type-2 diabetes, Metformin decreased TGF-β1 and macrophage infiltration in UUO-OKs, showing lower levels of collagen and α-SMA. It activated AMP-activated protein kinase (AMPK) and likely suppressed TGF-β1 to decrease fibrosis and inflammation [23].

#### PR-619

A pan-deubiquitinating enzyme inhibitor, PR-619 blocked fibrosis in UUO-OKs by promoting protein degradation and disrupting the TGF-β1 signaling, particularly Smad4 protein, which is essential for fibrosis-related transcription [24].

#### Telbivudine

An antiviral for hepatitis B, Telbivudine was effective in UUO-OKs, reducing fibrosis markers and proinflammatory cytokines (TNF-α). It affected NF-κB pathway, indicating it could improve renal function in patients with kidney disease [25].

### Stem cell therapy

Stem cell therapy shows great potential in healing kidney injuries. Research by Sun et al. [26] explored the impact of transplanting human amniotic fluid-derived stem cells (hAFSCs) into damaged renal tissues. In cases treated with hAFSCs, there was a noticeable reduction in kidney damage and a decrease in the expansion of the interstitial area. Treatment also helped maintain peritubular capillary density, a vital component of healthy kidney structure. Levels of VEGF and E-cadherin proteins, which support vascular integrity and cellular adhesion, were elevated in treated cases compared to untreated ones. Additionally, treatment with hAFSCs significantly lowered the levels of proteins associated with kidney injury and inflammation, such as HIF-1α, collagen I, TGF-β1, and MCP-1. Notably, the hAFSCs treatment promoted tubular cell proliferation at day 14 of injury and reduced the presence of apoptotic cells in the renal tubules. This finding is unique, as hAFSCs treatment was the only one in the study to enhance tubular epithelial cell proliferation, making it a promising therapy for kidney regeneration and highlighting the need for further investigation into its mechanisms and long-term effects.

Recent research suggests that small extracellular vesicles (sEVs) derived from stem cells may play a role in preventing and even reversing fibrosis by promoting tissue repair and reducing inflammation. However, challenges in isolating, dosing, and delivering sEVs for clinical use must be addressed before they can be considered a viable therapy for renal fibrosis. Developing a cure for renal fibrosis remains a significant challenge, particularly because the disease is often detected after renal dysfunction has already set in. The complexity of fibrosis, involving multiple signaling pathways and cellular interactions, makes it difficult to reverse once established [27]. Moreover, while stem cell therapy has shown regenerative potential, its mechanisms and long-term efficacy require further study.

### Zinc oxide nanoparticles

Administration of a single nontoxic dose (5 mg/kg) of ZnONPs ase recommended by Barakat et al. [28] significantly improved kidney structure and function after 14 and 30 days of PUUO induction.. Tian et al. [29] found that ZnONPs significantly mitigated the PUUO-induced renal injury suppressing of oxidative stress (MDA and TOS), upregulating of antioxidant (SOD) and antiapoptotic genes (BCL2), and downregulating the expression of inflammatory cytokines (TNF-α, and IL6), apoptotic gene (Bax) and fibrotic marker (β-Catenin. ZnONPs prevent cell apoptosis by increasing the mitochondrial function and decreasing the release of apoptosis-inducing factors and cytochrome c that cause internal signal apoptosis.

In a study by Khater et al. [30]declared that despite advances in supportive precautions and preventive strategies, there is currently no specific medication in clinical use for PUUO-induced renal damage.They suggested that adding ZnONPs to losartan could have synergistic renoprotective against PUUO-induced chronic renal cascades through improvement the renal function tests, amelioration of oxidative stress, inhibition of induced apoptosis and fibrosis with marked renal regeneration which highlights the possible application of these drugs as a complementary therapies for different chronic renal degenerative diseases.

## Conclusion

Untreated, ureteral obstruction can cause irreversible renal damage marked by fibrosis and functional decline. While relieving the obstruction (RUUO) is essential to prevent further injury, studies indicate that RUUO alone may not fully restore renal function and structure, particularly after prolonged obstruction. Complementary pharmacological treatments could be crucial in supporting renal recovery by mitigating oxidative stress, inflammation, and fibrosis.

## Data Availability

No datasets were generated or analysed during the current study.
